# Metal Ions in Polydopamine Coatings Enhance Polymer–Metal Adhesion

**DOI:** 10.1021/acsapm.4c03551

**Published:** 2025-02-12

**Authors:** Georgios Kafkopoulos, Ricardo P. Martinho, Clemens J. Padberg, Joost Duvigneau, Frederik R. Wurm, Gyula Julius Vancso

**Affiliations:** † Department of Materials Science and Technology of Polymers (MTP), University of Twente, Enschede 7522 NB, The Netherlands; ‡ Sustainable Polymer Chemistry (SPC), Department of Molecules and Materials, MESA+ Institute for Nanotechnology, Faculty of Science and Technology, University of Twente, Enschede 7522 NB, The Netherlands; § Department of Molecules and Materials, MESA+ Institute for Nanotechnology, Faculty of Science and Technology, University of Twente, Enschede 7500 AE, The Netherlands

**Keywords:** polydopamine, metal ion, polymer metal adhesion, PC, titanium, ^13^C solid-state NMR

## Abstract

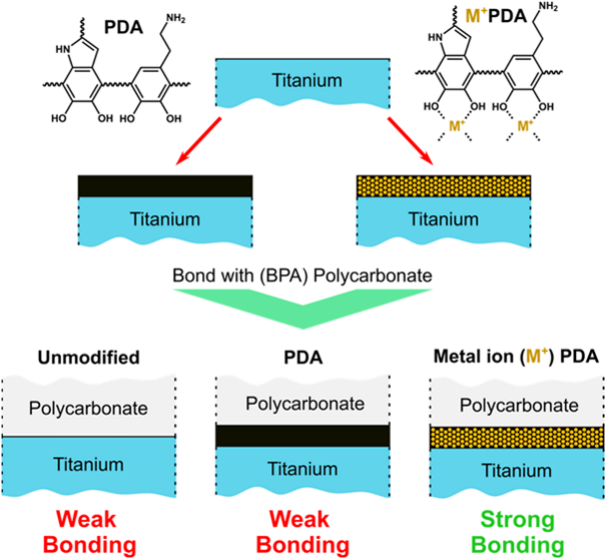

Strong bonding at polymer–metal oxide interfaces is of high importance for lightweight thermoplastic composite structures. However, interfacial adhesion in polymer–metal material systems often poses grand challenges in applications, and hence, tailoring the molecular interactions is necessary. Here, the interfacial adhesion between polycarbonate (PC) and titanium (Ti) is optimized by utilizing metal ion-containing polydopamine (M^+^PDA) produced using two methods, i.e., postdeposition and codeposition with respect to the PDA polymerization. M^+^PDA thin films were formed on the surface of titanium wires, which were then comolded with a PC matrix to form pullout samples in order to evaluate the interfacial energy of adhesion (*G*_a_). For the postdeposition process, Fe^3+^-, Fe^2+^-, Co^2+^-, Ni^2+^-, Cu^2+^-, or Zn^2+^-containing PDA layers were evaluated. Fe^3+^PDA and Fe^2+^PDA coatings resulted in a significant increase of *G*_a_ between Ti and PC, while the other metal ions had an insignificant effect. For the codeposition process, Cu^2+^ was utilized, and CuPDA films of various Cu^2+^:DA ratios were evaluated. *G*_a_ values for these systems followed an increasing trend by increasing the Cu^2+^:DA ratio until a plateau was reached at a 1:1 value. The M^+^ content had no influence on the values of *G*_a_, regardless of the deposition process utilized. In addition to the specific results to obtain strong adhesion at PC-Ti interfaces, by FTIR, AFM, and solid-state NMR, we also provide insights into hitherto unknown features regarding the surface morphology and chemistry of M^+^-containing PDA films.

## 1 Introduction

The use of polydopamine (PDA) is a versatile, simple and cost-effective surface modification method applicable to virtually any type of substrate.^1^ Since its first introduction in 2007,^2^ PDA has been successfully applied in many applications ranging from biomedical and energy-related fields, to polymer composites.^3,4^ We have recently highlighted the potential of PDA to transfer load at polymer metal interfaces joined at elevated temperatures.^5−7^ This effect is attributed to two main factors, i.e., the thermal transformation of PDA upon heating^8^ and the tunability of surface chemistry^9^ by PDA derivatives. The thermal transformation of PDA has been shown to enhance its mechanical performance,^10^ thus high processing temperatures are expected to result in more robust coatings. In terms of molecular bonding, there are material systems such as PMMA and titanium,^11^ where PDA cannot bond with the adherent during the joining process due to lack of reactive sites. In such cases a series of modification techniques can be applied during or after the formation of PDA,^12^ allowing one to tune the chemistry with the aim to promote molecular interactions.

An attractive and widely applied method to functionalize PDA is the incorporation of metal ions (M^+^) resulting in metal ion-containing polydopamine (M^+^PDA).^13^ The presence of a variety of M^+^ in PDA is reported to have a positive impact on the mechanical properties^14^ and stability^15^ of the coatings. The improved properties have been attributed to the coordination of catechol moieties of PDA with the respective M^+^, resulting in coordinative cross-linking of the coatings. In addition, the presence of M^+^ in PDA provide additional functionalities, such as improved electrical conductivity^16^ as well as antibacterial^17^ and catalytic activity.^18^ M^+^ incorporation in PDA can be achieved either by polymerizing dopamine (DA) in the presence of M^+^,^19^ i.e., codeposition, or by immersing PDA in M^+^ aq. solutions to absorb the M^+^,^15^ i.e., postdeposition. In this work M^+^PDA coatings produced by both processes were utilized in polymer–metal interfaces aiming for load transfer in composite applications.

We focus here on the promoting of strong bonding at titanium oxide–polycarbonate interfaces, which is of relevance for the mechanical performance of advanced materials ranging from nanocomposites^20^ to hybrid structures.^21^ M^+^PDA coatings were formed on the surface of titanium (Ti), which are then joined via a comolding process with a bisphenol A polycarbonate (PC). Interfacial adhesion between the M^+^PDA surface modified Ti and PC is determined by fiber pullout testing. The M^+^PDA coatings under consideration were produced with the postdeposition process using Fe^3+^, Fe^2+^, Co^2+^, Ni^2+^, Cu^2+^ or Zn^2+^. In addition, in order to evaluate the effect of M^+^ content of the M^+^PDA on the PC-Ti interfacial adhesion, coatings were formed via the codeposition process using Cu^2+^. The objective of this study is to identify the effect of the deposition process and the choice of the ions on the morphology, chemistry and M^+^ content of M^+^PDA coatings and correlate it to the PC-Ti interfacial adhesion.

## 2 Experimental Section

### 2.1 Materials

Tris(hydroxymethyl)-aminomethane buffer (TRIS), *N,N*-Bis(2-hydroxyethyl) glycine buffer (Bicine), dopamine hydrochloride, iron(III) chloride (FeCl_3_, 97%), iron(II) chloride (FeCl_2_, 98%), cobalt(II) chloride (CoCl_2_, 97%), nickel(II) chloride (NiCl_2_, 98%), copper(II) chloride (CuCl_2_, 97%,) and zinc(II) chloride (ZnCl_2_, 98%) were purchased from Sigma-Aldrich (Zwijndrecht, The Netherlands) and were used as received. Toluene, acetone and 2-propanol were purchased from VWR (Amsterdam, The Netherlands). Titanium IV oxide, anatase, nanoparticles (99,7%, diameter <25 nm). Bisphenol A polycarbonate (PC) granules, Makrolon 2408, were kindly provided by Covestro A.G. (Leverkusen, Germany). Titanium grade 5 (Ti6Al4 V) wires of 1 mm diameter, a surface roughness (*R*_a_) of 1.3 μm and a modulus (*E*_f_) of 110 GPa and were purchased from SELFAN Fine + Metal GmbH (Köln, Germany).

### 2.2 Polydopamine and Metal Ion Polydopamine Deposition on the Surface of Titanium Wires

Wire specimens were cut to 9 cm long pieces, polished, solvent cleaned and dried according to our previous protocol.^7^ These wires from now on will be referred to as “Ti”. Layers of polydopamine (PDA), metal ion loaded polydopamine using a postdeposition process (M^+^@PDA) or copper-polydopamine using a codeposition process (CuPDA) were formed on the surface of Ti wires according to the procedures described below.

**Figure 1:**
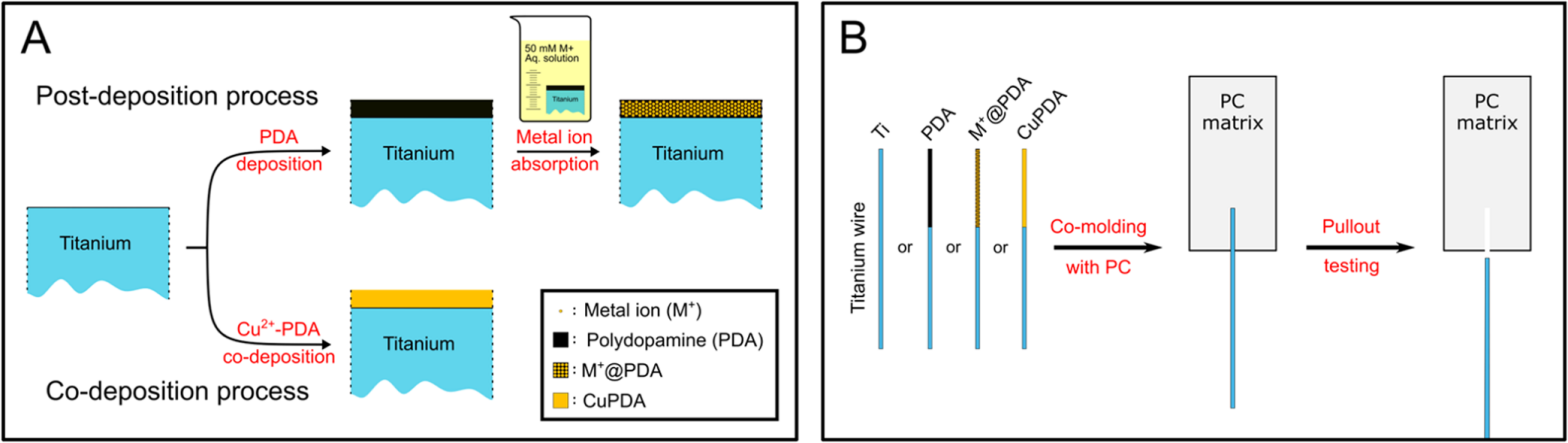
Schematic representation of PDA, M^+^@PDA postdeposition and CuPDA codeposition film formation processes on titanium (A); M^+^ stands for Fe^3+^, Fe^2+^, Co^2+^, Ni^2+^, Cu^2+^ or Zn^2+^. Schematic representation of comolding and testing of pullout specimens prepared using polycarbonate and PDA, M^+^@PDA or CuPDA-coated titanium wires (B).

PDA layers were formed on titanium wires by dip coating (Figure 1A). In a typical process, 6 Ti wires were treated with O_2_ plasma (Plasma Prep II SPI; West Chester) for 1 min using a current of 40 mA and a pressure of 0.27 mbar. The plasma treated wires were then immersed in a freshly prepared 10 mM TRIS buffer aqueous solution containing 2 mg/mL dopamine hydrochloride (DA). The wires were then left in the solution in ambient conditions for 24 h to form a PDA layer on their surface. The PDA coated wires were then removed from the solution, washed thoroughly with milli-Q water (Milli-Q Advantage A10, Millipore) and dried under vacuum at 40 °C overnight. The PDA coated wires from now on will be referred to as “PDA”. For the purpose of a control experiment, PDA coatings were also deposited onto Ti wires in a similar manner by using 2 mg/mL DA in 50 mM TRIS or 100 mM bicine buffer solutions. For the PDA deposition using bicine solution the pH was set to 8.5 using a 2 M sodium hydroxide (NaOH) solution.

M^+^@PDA coatings on Ti wires in the postdeposition process were obtained by immersing 6 PDA coated wires in a 50 mM metal ion chloride aqueous solution for 1 h. Then the wires were removed from the solution, washed thoroughly with milli-Q water and dried under vacuum overnight. The M^+^@PDA coated wires obtained by postdeposition from now on will be referred to as “M^+^@PDA”, with M^+^ to be Fe^3+^, Fe^2+^, Co^2+^, Ni^2+^, Cu^2+^ or Zn^2+^.

CuPDA layers were formed on titanium also by dip coating. Aqueous solutions were prepared using 2 mg/mL DA and different copper(II) chloride (CuCl_2_) concentrations. The copper:dopamine molar ratios used were 1:8, 1:4, 1:2, 1:1, 2:1 and 3:1. The pH of the solutions was then tuned to 8.5 by using a 2 M TRIS buffer aqueous solution followed by the immersion of 6 O_2_ plasma treated Ti wires into each solution. After 24 h the CuPDA-coated wires were removed from the solution, washed thoroughly with milli-Q water and dried under vacuum overnight. As a control experiment, CuPDA coatings were deposited onto Ti wires in a similar manner by using 2 mg/mL DA in a 100 mM bicine buffer solution. The pH was adjusted to 8.5 using a 2 M sodium hydroxide (NaOH) solution.

### 2.3 Polycarbonate-Titanium Pullout Sample Preparation and Testing

Polycarbonate-titanium pullout samples were prepared by a comolding process. PC granulates were dried under vacuum at 60 °C overnight and were then compression molded with Ti, PDA, M^+^@PDA and CuPDA wires using a stainless-steel mold and a THB 400 press (Fontijne, Delft, The Netherlands). For details on the mold design and preparation process before compression molding we refer to our previous work.^11^ Compression molding was performed at 200 °C under a pressure of 40 MPa for 5 min followed by cooling to room temperature. The pullout specimens comprised of a titanium wire embedded ∼5 mm in a PC block (40 × 20 × 4 mm^3^). After the demolding process the pullout samples were stored under vacuum until the pullout testing process took place. The pullout test specimens were mounted on a tensile testing apparatus (Zwick i-line Z5.0, Zwick/Roel, Ulm, Germany) using modified tensile grips,^11^ and were then subjected to the tests at a cross-head speed of 10 mm min^–1^. A minimum of 6 samples were tested per sample category. The comolding and pullout testing process are schematically summarized in Figure 1B.

### 2.4 Characterization Methods

X-ray photoelectron spectroscopy (XPS; PHI Quantes, Physical Electronics) was employed to quantify the atomic concentrations in PDA, M^+^@PDA and CuPDA layers deposited on Ti wires. The measurements were performed at a chamber base pressure and working pressure of 7 × 10^–7^ Pa and 1.3 × 10^–6^ Pa, respectively (Argon atmosphere). A monochromatic Al Ka X-ray source was utilized at 1486.6 eV with a 50 μm diameter beam, 12.5 W X-ray gun power and a beam/detector input angle of 45°.

Atomic force microscopy (AFM, MultiMode 8, HarmoniX, NanoScope V controller, JV vertical engage scanner, Bruker, Santa Barbara) was used to evaluate the effect of the postdeposition process using different metal ions on the surface morphology of M^+^@PDA coatings. In a typical process PDA was deposited on a ∼6 × 2 cm^2^ silicon oxide wafer coated with a ∼60 nm thick Ti layer (Ti@SiO_2_). The PDA deposition was conducted under vigorous stirring using 2 mg mL^–1^ DA in a 10 mM TRIS aqueous (Milli-Q) solution, after which washing and drying steps were applied (see section 2.2). The PDA coated Ti@SiO_2_ wafer was then cut into 1 × 2 cm^2^ pieces, each of which was used to study each M^+^ ion, i.e., Fe^3+^, Fe^2+^, Co^2+^, Ni^2+^, Cu^2+^ or Zn^2+^, using the following methodology. The 1 × 2 cm^2^ Ti@SiO_2_ wafer was cut in two 1 × 1 cm^2^ pieces, of which one was used as a reference and the other was immersed in a 50 mM M^+^ aqueous solution for 1 h, followed by washing and drying (see section 2.2). AFM was then conducted on the surface of the reference as well as of the immersed sample, at close proximity to the cutting point used to separate the two wafers before immersion. This procedure ensured minimizing uncertainties related to surface morphology variations at different locations of the PDA coating. The AFM measurements were conducted in the Peak Force Quantitative Nanomechanical Mapping mode (PF-QNM). A silicon AFM probe (Tap150Al-G) was used with a nominal tip radius, resonance frequency and spring constant of 10 nm, 150 kHz and 5 N m^–1^, respectively. The AFM optical sensitivity was calibrated using the thermal tuning method and the data were analyzed using the NanoScope Analysis software (version 1.9).

Scanning electron microscopy (SEM, JSM 7610 FPlus; JEOL) was employed as a complementary method to evaluate the effect of the postdeposition process on the surface morphology using different metal ions, as well as to determine the thickness of M^+^@PDA coatings. Reference PDA and M^+^@PDA coated Ti@SiO_2_ wafers were prepared using the same methodology with the samples prepared for AFM. The PDA and M^+^@PDA coated Ti@SiO_2_ wafers were cut using a diamond pen and were mounted on a holder for cross-sectional SEM analysis. In SEM experiments, the working distance (W.D.) was 8 mm and the acceleration voltage was 1.5 kV.

Fourier transform infrared (FTIR) spectroscopy was employed to provide insights on the effect of copper codeposition and type/concentration of buffer on the final chemical structure of PDA. FTIR measurements were performed on CuPDA and PDA precipitates formed in the polymerization solutions where the titanium wires were immersed for PDA deposition. The precipitates were isolated from their respective post polymerization solutions with five consecutive centrifugation steps (Z36HK, HERMLE Labotechnik, Germany) and milli-Q washing cycles, and then dried overnight under vacuum at 40 °C. FTIR-ATR (α, Bruker, Leiderdorp, The Netherlands) spectra were obtained from 4000 to 400 cm^–1^ with a resolution of 4 cm^–1^, averaged over 64 scans. The spectra were subjected to a 25-point smoothening and then a baseline correction.

Solid-state NMR experiments were performed on PDA and CuPDA-coated titanium nanoparticles to verify the formation of chemical species in CuPDA coatings, not present in PDA. PDA and CuPDA-coated titanium nanoparticles were obtained by ultrasonically dispersing 4 mg mL^–1^ Ti nanoparticles in 100 mL milli-Q water. The dispersion was then subjected to vigorous stirring followed by the addition of dopamine hydrochloride, buffer (TRIS or bicine) and copper chloride (see section 2.2 for the concentrations/protocol of each sample category). The suspension was left to polymerize for 24 h under vigorous stirring. The coated titanium nanoparticles were then separated from the aqueous solutions by letting them sediment, redisperse them in milli-Q water, a total of 5 times, followed by drying under vacuum at 40 °C overnight. The samples were ground resorting to a mortar and pestle and packed tightly in a rotor for solid-state NMR. The measurements were performed using a Bruker Avance Neo spectrometer operating a 14.1 T magnet (with 600.16 MHz for 1H and 150.93 MHz for 13C) equipped with a 2.5 mm magic angle spinning (MAS) probe. 13C Cross-Polarization (CP) MAS measurements were carried out at a spinning rate of 15 kHz, with a 90° pulse for 1H of 2.25 μs and a contact time of 2 ms, and a minimum of 14336 averages. For samples containing only diamagnetic species a recycle delay of 5 s was employed, while those with paramagnetic species (Copper), measurements were conducted using a 0.5 s recycle delay, given their shorter relaxation times. 13C spectra were referenced externally to adamantane and acquired with 2272 complex points with a spectral width of 301 ppm (45.5 kHz). All NMR spectra were zero-filled, apodized, subject to zeroth order phasing, and Fourier transformed using MestReNova.

## 3 Results and Discussion

### 3.1 Pullout Testing to Evaluate the PC-Titanium Interfacial Adhesion

We utilize pullout testing of titanium (Ti) wires from a polycarbonate (PC) matrix to evaluate the potential of metal ion-containing PDA coatings to promote adhesion at PC-Ti interfaces. Adhesion is discussed in terms of the interfacial energy of adhesion (*G*_a_), which is calculated by analyzing the resulting pullout curves. For more details on the methodology used we refer to the Supporting Information (SI-1) for PC, or to our previous reports for PMMA^11^ and PLA^5^ matrices.

### 3.2 Post-Deposition of M^+^@PDA

**Figure 2:**
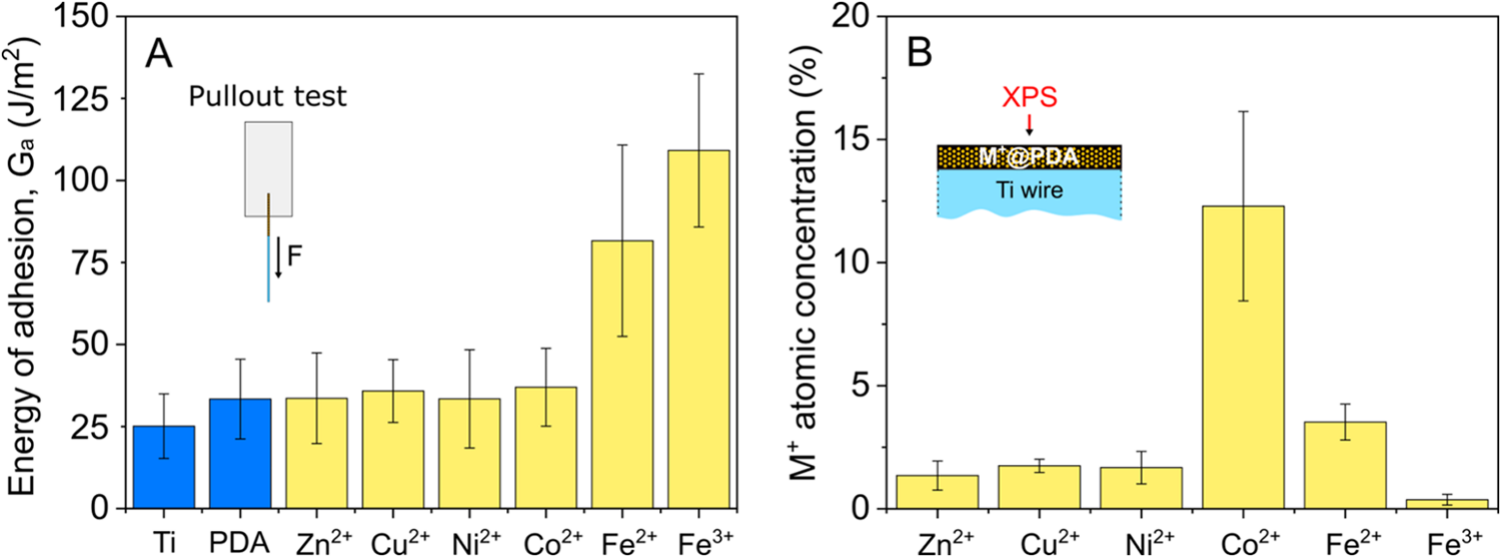
Energy of adhesion values between PC and unmodified, PDA or M^+^@PDA coated titanium wires, determined with pullout tests (A); the blue bars represent the *G*_a_ values for unmodified and PDA coated Ti and are highlighted for comparison. Metal ion respective % atomic concentration of M^+^@PDA coatings on titanium wires determined with XPS (B).

PDA layers were formed on titanium wires, which were immersed in aqueous solutions of the respective metal salts (M^+^, 50 mM), i.e., Fe^3+^, Fe^2+^, Co^2+^, Ni^2+^, Cu^2+^ or Zn^2+^. The resulting M^+^@PDA coated wires were then embedded in a PC matrix and were subjected to pullout testing. The energy of adhesion values (*G*_a_) between PC and unmodified, PDA or M^+^@PDA wires are shown in Figure 2A. The respective pullout plots and the values used to determine *G*_a_ can be found in the Supporting Information (SI-2). By introducing a PDA-layer on the surface of titanium, a slight increase in G_a_ was observed compared to the bare titanium surface. A similar increase was observed for M^+^@PDA coatings, in which M^+^ was Co^2+^, Ni^2+^, Cu^2+^ or Zn^2+^. M^+^@PDA coatings containing Fe^2+^ and Fe^3+^, exhibited a 3-fold and 4-fold increase in their *G*_a_ values, respectively, when compared to the unmodified titanium surface. In previous works we have showcased the excellent ability of PDA to transfer load at interfaces, which was accounted to the strong bonding of PDA layers on titanium surfaces in dry conditions as well as their robust mechanical properties after processing at elevated temperatures.^6,7^ Thus, we interpret the G_a_ value increase when using Fe^2+^PDA and Fe^3+^PDA coatings as an increased bonding between the PDA layer and the PC matrix.

The origins of the strong bonding of Fe^2+^PDA and Fe^3+^PDA coatings with PC remains an open question. Under the assumption that metal ions are incorporated in the PDA layers without changing the chemical structure of PDA, we hypothesize that two factors could explain our observations. The first is that the incorporated Fe^2+^ and Fe^3+^ ions promote bonding between PDA and PC either via coordination bonds (i.e., dative covalent bonds) or by acting as catalysts that activate reactions between PDA and PC during the comolding process. The second factor is related to how the M^+^ incorporation process affects the surface morphology of the coatings. Alteration of the surface morphology could potentially result in stronger bonding between PC and PDA either by increasing the surface contact area or by promoting morphological interlocking between adherent and adhesive. Hence our goal now is to attempt elucidating to what extent these two factors are related to the observed strong bonding between PDA and Fe^2+^PDA/Fe^3+^PDA coatings.

**Figure 3:**
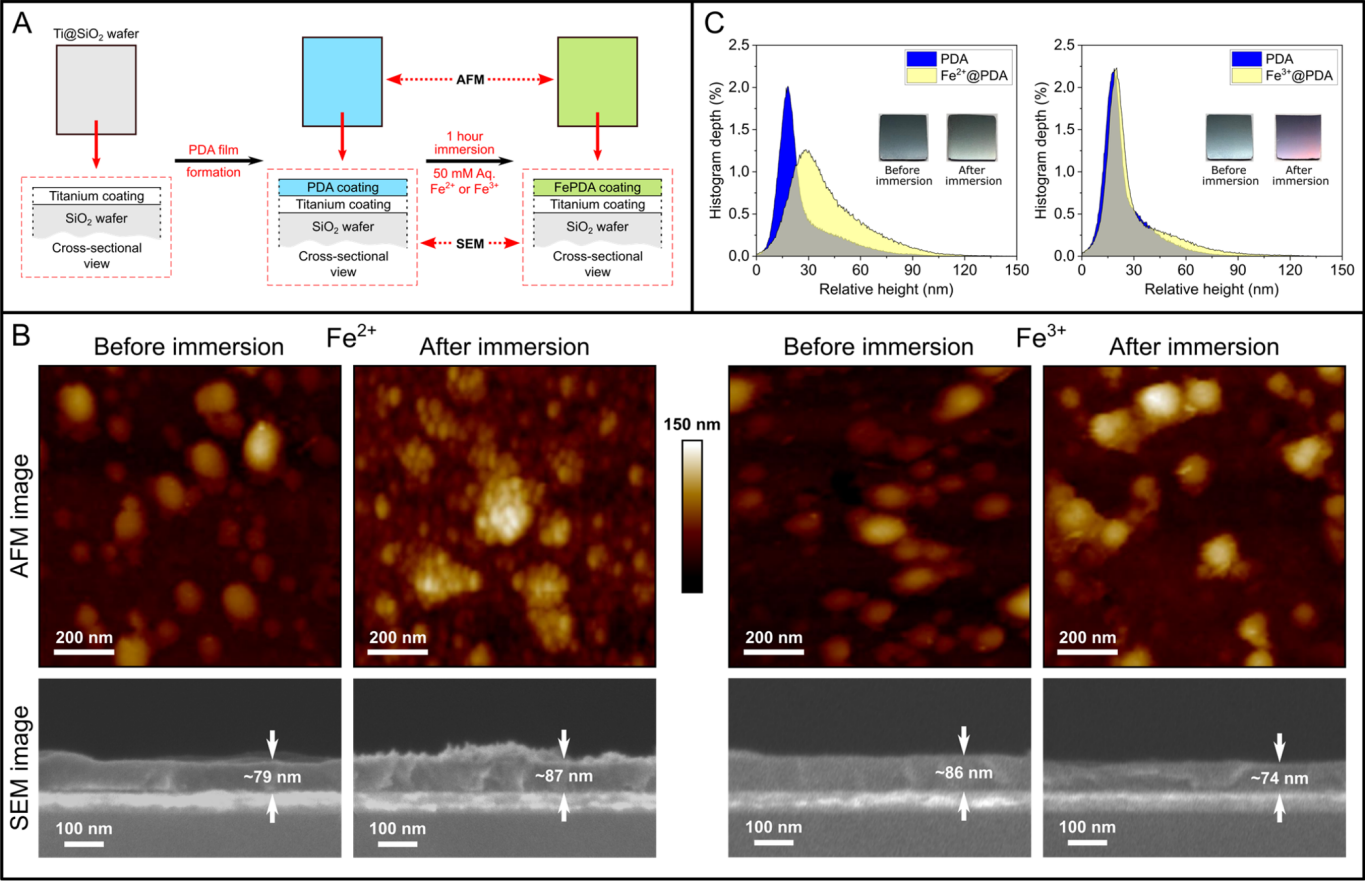
(A) Schematic of PDA/M^+^@PDA deposition and AFM/SEM imaging procedures. (B) AFM surface topography images and SEM cross-sectional images of Fe^2+^PDA and Fe^3+^PDA coatings deposited on Ti@SiO_2_ wafers before and after immersion in their respective metal ion solutions; the dashed rectangles in the AFM topography images indicate the area where the inset magnified images originate from. (C) Relative height distribution of the surface of Fe^2+^PDA and Fe^3+^PDA coatings before and after immersion in their respective metal ion solutions; the height values are with respect to the lowest surface point of each coating; the insets are photographs taken in an angle of ∼30° of the PDA coated Ti@SiO_2_ wafers before and after immersion in the metal ion solutions.

AFM was employed to evaluate whether the metal ion incorporation process affects the surface morphology of the PDA coatings. AFM imaging was conducted on the surface of PDA coatings deposited on Ti@SiO_2_ wafers before and after immersion in 50 mM M^+^ aqueous solutions. A schematic representation of the experimental procedure is shown in Figure 3A. The surface morphology and thickness of the PDA coatings immersed in Co^2+^, Ni^2+^, Cu^2+^ or Zn^2+^ aqueous solutions remained unchanged and thus these results are only shown in the Supporting Information (SI-3). Figure 3B shows the AFM surface topography and cross-sectional SEM images of Fe^2+^PDA and Fe^3+^PDA coatings before and after immersion in their respective iron ion solutions. Before immersion, the PDA coatings consist of smooth regions and “hills”, with the latter to be PDA particles incorporated in the PDA films during the deposition process.^22^

PDA coatings immersed in a 50 mM Fe^2+^ aqueous solution exhibit a peculiar change in their surface morphology. AFM images (Figure 3B) show the formation of “raspberry” like structures on the surface of the PDA layer after the immersion process. The change in the surface morphology can also be visualized by plotting the surface height distribution (Figure 3C), where a notable shift and broadening of the distribution curve is observed after the immersion process. Cross-sectional SEM images (Figure 3B), apart from a slight thickness increase, also indicate that the structures formed on the surface of PDA are “spikes” rather than “raspberries”. From the morphological features of the coatings identified with AFM and SEM, as well as the comparatively increased O content of the particular films identified with XPS measurements (Supporting Information, SI-4), we propose that the observed features are likely FeO_*x*_ structures formed on the surface of PDA upon the oxidation of Fe^2+^.To further support our hypothesis, we note that the formation of nanofeatures on the surface of PDA have been also observed when post depositing palladium, silver or gold ions in PDA layers.^23,24^ Overall immersing PDA layers in a 50 mM Fe^2+^ aqueous solution results in a significantly altered surface morphology with a more rough surface.

AFM imaging and data analysis (Figure 3B,C) show that PDA coatings before and after immersion in a 50 mM Fe^3+^ aqueous solution do not exhibit significant surface morphology differences. SEM imaging (Figure 3C), however, shows a notable thickness reduction of the coatings after the immersion process, from roughly 86 to 74 nm. This thickness reduction is also reflected by the surface color change of the coated substrates, which changed from light blue to violet (insets in Figure 3C). This color change is in agreement with what has been previously reported for PDA coated substrates with layers of varying thicknesses.^25^ The thickness reduction could be the result of two processes. The first process involves etching of the PDA coatings during the immersion process. The second possibility includes dehydration of the coatings by water displacement caused by the incorporation of Fe^3+^ ions which are assumed to cross-link PDA by acting as coordination centers for the catechol moieties.^13^ Overall, immersing PDA layers in a 50 mM Fe^3+^ aqueous solution does not cause significant surface morphology alterations, but rather a thickness reduction of the coatings.

Overall, the M^+^ post deposition process does not impact the surface morphology of PDA coatings for Fe^3+^, Co^2+^, Ni^2+^, Cu^2+^ and Zn^2+^, while a significant change is observed for Fe^2+^. The adhesion tests showed that only Fe^2+^PDA and Fe^3+^PDA layers resulted in a *G*_a_ value increase between titanium and PC when used as adhesive interlayers. The rest M^+^@PDA coatings did not exhibit a significant impact on *G*_a_. Assuming that M^+^@PDA coatings deposited on the surface of titanium wires are analogous to the ones deposited on Ti@SiO_2_ wafers, the surface morphology could only potentially explain the enhanced adhesion for Fe^2+^PDA. This denotes that Fe^3+^PDA potentially bonds with the PC melt via coordination bonds or by catalytically activated covalent bond formation during the comolding process. The latter could be supported by previously reported melt transesterification reactions between polycarbonate and hydroxyl containing benzophenone derivatives using iron chloride as a catalyst.^26^ Additionally, aminolysis melt-reactions between primary amines and carbonate groups in PC are also reported,^27^ however in this work no catalyst was applied. Thus, it could be assumed that the enhanced adhesion is the result of bonds formed between Fe^3+^PDA and PC via Fe^3+^ catalyzed transesterification or aminolysis reactions between catechol groups or primary amines in PDA and carbonate bonds in PC.

Since the presence of Fe^3+^ ions in PDA likely favors the formation of covalent bonds between PC and PDA, we focus on parameters that could help to elucidate the bonding mechanism. Such a parameter could be the amount of metal ions incorporated in each of the applied M^+^@PDA coatings. Figure 2B shows the % M^+^ atomic concentration (*C*_a_) in the respective M^+^@PDA coatings deposited on the surface of titanium wires. The concentrations (as determined using XPS and the spectra/atomic concentration values) can be found in the Supporting Information (SI-4). Ni^2+^, Cu^2+^ and Zn^2+^ were incorporated in PDA in similar atomic concentrations of ∼1.5%. Fe^2+^ content in Fe^2+^PDA was found to be ∼3.5%, however the potential formation of FeO_*x*_ spikes on the surface of PDA renders the measured values for Fe^2+^ incomparable with the rest of the M^+^@PDA coatings. Co^2+^ exhibited the highest metal ion content, i.e., ∼12% and Fe^3+^ the lowest, i.e., ∼0.3%. Overall, no correlation is observed between the *G*_a_ values and the M^+^ atomic concentrations of the respective M^+^@PDA coatings.

The lack of correlation between *G*_a_ and M^+^ concentration of M^+^@PDA coatings is not highly surprising, as more parameters related to the choice of M^+^ can also be important. These parameters include valency, complexation strength, ligand bonding geometry or catalytic activity. Thus, the M^+^ atomic concentration should be considered as a parameter for only one type of M^+^ to evaluate its effect on the adhesive performance of M^+^@PDA coatings. However, the postdeposition process of M^+^ in PDA has been shown to be inefficient to result in high M^+^ loadings as well as provide good control over the M^+^ loading.^13^ Hence in the following section we employ a different process to attempt the production of M^+^@PDA coatings with higher M^+^ loadings,^13^ i.e., the codeposition process. However, under the applied experimental conditions, trials revealed no PDA film formation and growth (detectible by SEM) on titanium for all M^+^ but Cu^2+^. This resulted in no improvement in terms of interfacial bonding for all M^+^PDA films but CuPDA (see SI-5). Hence, for the codeposition process only CuPDA films with various Cu^2+^ loadings were considered.

### 3.3 Codeposition of Cu^2+^ and Dopamine

**Figure 4:**
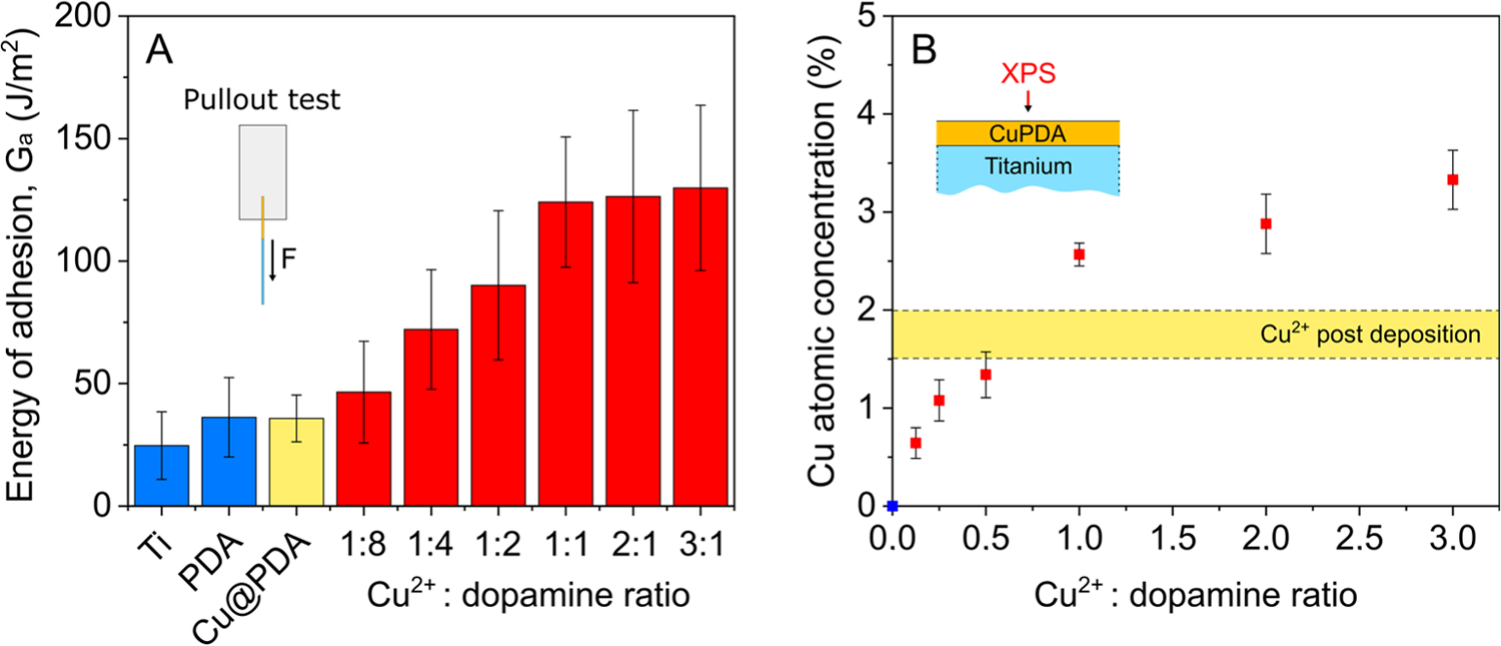
Energy of adhesion values between PC and unmodified, PDA, Cu^2+^@PDA and CuPDA-coated titanium wires, determined by pullout tests (A). Metal ion % atomic concentration of CuPDA coatings on titanium wires determined by XPS versus the Cu^2+^:DA ratio used to produce the coatings (B). XPS spectra and determined atomic concentrations can be found in (SI-7).

To evaluate the effect of M^+^ loading of M^+^PDA on the *G*_a_ values between titanium and PC, was investigated using Cu^2+^. Titanium wires were coated with CuPDA using a codeposition process. This process is similar to the conventional PDA deposition^2^ differing only that CuCl_2_ was added to the polymerization solution. The CuPDA-coated wires were then comolded with PC to form pullout specimens and determine the *G*_a_ values between titanium and PC. Figure 4A shows the *G*_a_ values between PC and unmodified, PDA-, Cu^2+^@PDA- and CuPDA-coated titanium wires. The respective pullout plots and the values used to determine *G*_a_ can be found in the Supporting Information (SI-6). By increasing the Cu^2+^:DA ratio, *G*_a_ values follow an increasing trend for ratios up to 1:1 and then plateaus. This trend is in close agreement with the trend observed for the Cu^2+^ loading of CuPDA coatings shown in Figure 4B. However, the Cu^2+^ loading of Cu^2+^@PDA coatings, is higher than CuPDA coatings with Cu^2+^:DA ratios from 1:8 to 1:2. The corresponding *G*_a_ values are however lower. This is an indication that the Cu^2+^ content does not have a major influence on the *G*_a_ values. Hence the codeposition process appears to influence the chemistry of CuPDA coatings more than just the incorporation of higher amounts of Cu^2+^.

**Figure 5:**
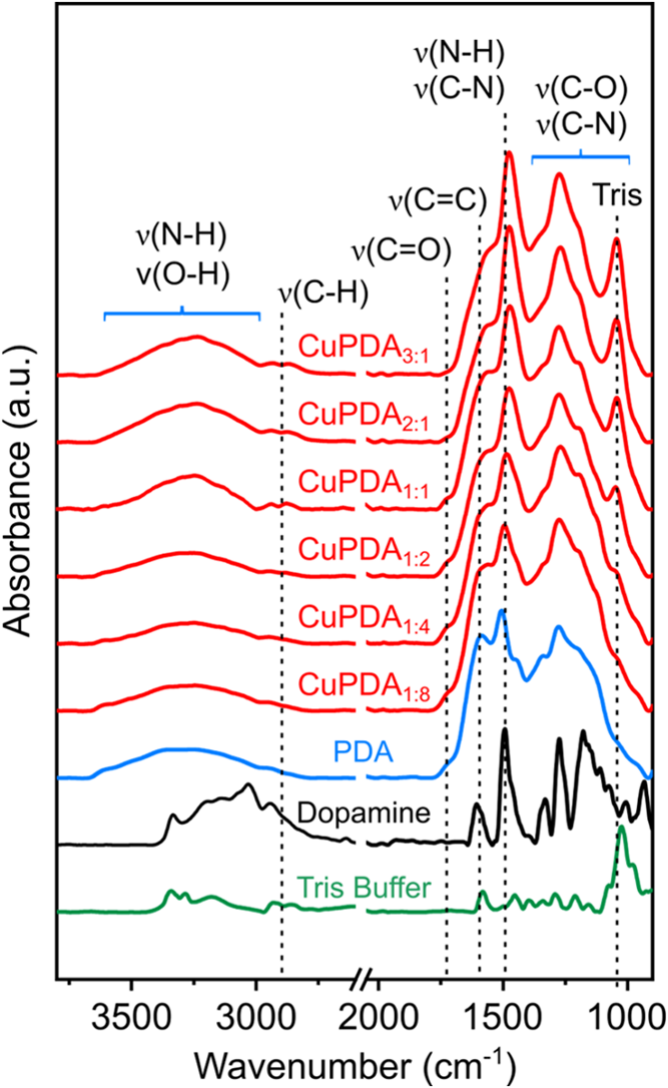
FTIR spectra obtained on DA, TRIS buffer, PDA and CuPDA precipitates. The CuPDA precipitates are the products of Cu^2+^:DA ranging from 1:8 to 3:1 using 2 mg mL^–1^ DA.

FTIR spectra obtained on PDA and CuPDA precipitates, isolated from the respective post polymerization solutions, are shown in Figure 5. The spectra reveal that the Cu^2+^:DA ratio has a significant impact on the chemical species present in the CuPDA coatings. The most notable change is observed at the peak intensity located at ∼1050 cm^–1^, which follows an increasing trend by increasing the Cu^2+^:DA ratio. This peak corresponds to the characteristic ν(C–O) stretching mode of the TRIS buffer.^28^ A higher Tris content used to regulate the pH at 8.5 at higher CuCl_2_ concentrations, also results in an increase of covalently bound TRIS in the coatings. The peak intensity at ∼1500 cm^–1^ corresponding to ν(C–N) stretching^29−31^ and ν(N–H) scissoring^29,32^ vibrations, also follows an increasing trend at higher Cu^2+^:DA ratio. Finally, the peak at ∼1300 cm^–1^ also show a gradual increase by increasing Cu^2+^:DA ratio. Assignment of the peaks at ∼1300 cm^–1^ is not feasible due to the overlapping of different peaks which are attributable to ν(C–O)^33−35^ and ν(C–N)^36^ vibrations. The increasing trend of absorbance at ∼1300 cm^–1^ can be perceived more easily when compared to the peak corresponding to the ν(C=C) stretching mode of the aromatic rings at ∼1585 cm^–1^.^35,37^

**Figure 6:**
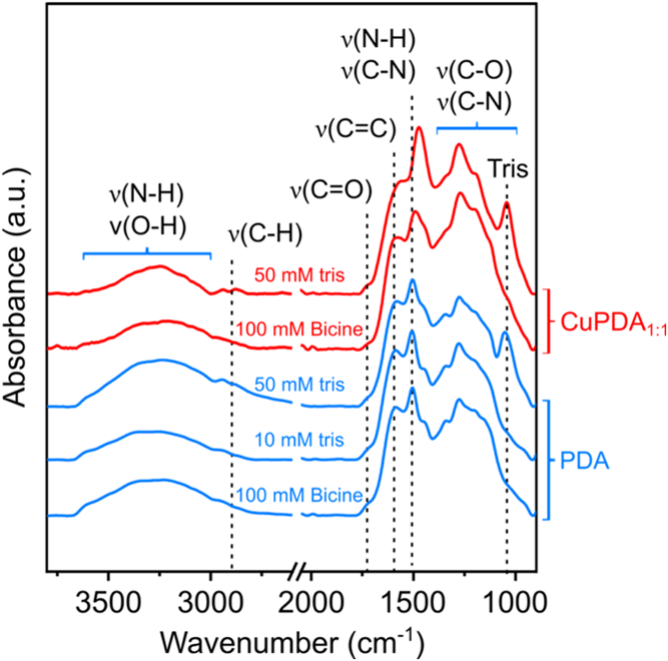
FTIR spectra obtained from PDA precipitates formed using 2 mg mL^–1^ dopamine and 10 mM TRIS, 50 mM TRIS or 100 mM bicine buffer and CuPDA precipitates formed using 50 mM TRIS or 100 mM bicine buffer.

From the analysis of the CuPDA FTIR spectra it is plausible that the *G*_a_ trend observed by increasing the Cu^2+^:DA ratio (see Figure 4A) could be caused by the incorporation of TRIS buffer. To test this hypothesis PDA and CuPDA_1:1_ coatings were formed on the surface of titanium wires using 50 mM TRIS or 100 mM bicine buffer. Bicine was chosen because it has been reported not to incorporate into PDA during the polymerization process.^1^ PDA precipitates formed using 10 mM TRIS and 100 mM bicine buffer show identical FTIR spectra (see Figure 6). The spectra of 50 mM TRIS only differs at the presence of the characteristic peak of TRIS at ∼1050 cm^–1^, which indicates the incorporation of TRIS to PDA. Similarly, in CuPDA_1:1_ precipitates formed using 100 mM bicine the characteristic peak of TRIS is not present when compared to CuPDA_1:1_ precipitates formed using 50 mM TRIS. We note that the Cu^2+^ incorporation in the films is also affected by the type of buffer, as 50 mM TRIS resulted in ∼2.6% while 100 mM bicine in ∼1% (see SI-8).

**Figure 7:**
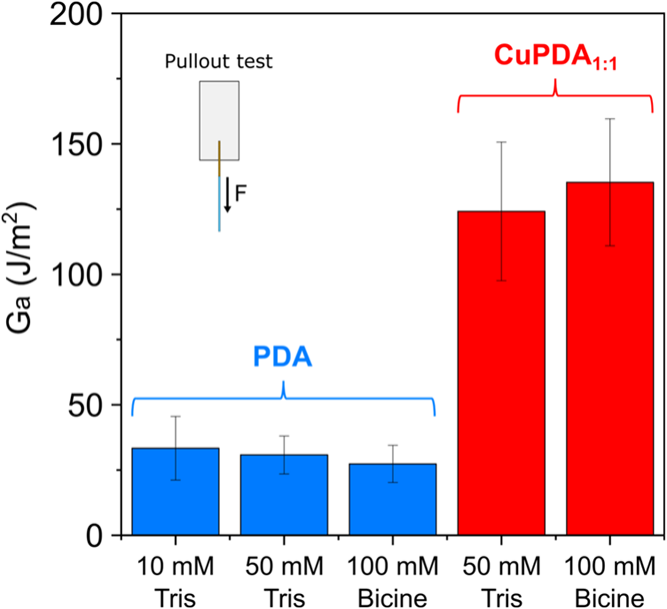
Energy of adhesion values between PC and PDA (10 mM TRIS, 50 mM TRIS or 100 mM bicine) or CuPDA (50 mM TRIS or 100 mM bicine) coated titanium wires, determined with pullout tests. The respective pullout plots and the values used to determine *G*_a_ can be found in SI-9.

Figure 7 shows the *G*_a_ values obtained using the aforementioned concentration/buffer formulations. The *G*_a_ values are similar for all PDA coatings as well as for CuPDA_1:1_. Thus, we can conclude that the incorporation of the TRIS buffer in the PDA coatings cannot explain the improved bonding caused by the CuPDA coatings at high Cu^2+^:DA ratios.

**Figure 8:**
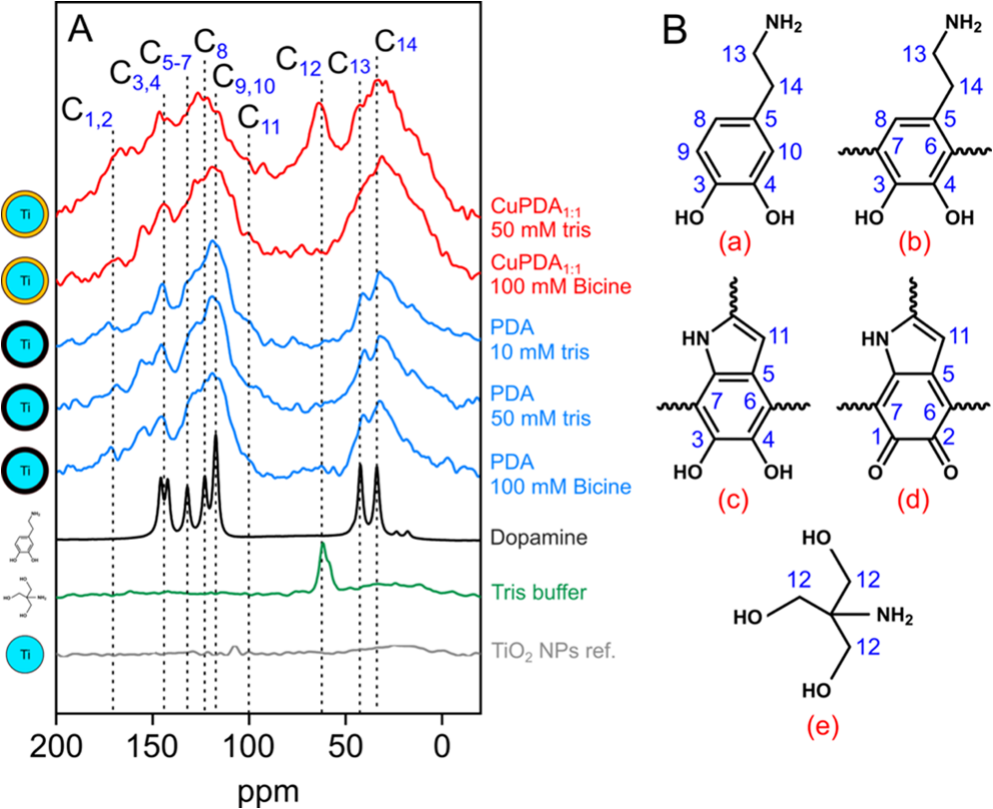
^13^C cross-polarization solid-state NMR spectra conducted under MAS conditions at 15 kHz obtained on the samples indicated on the margins. The dopamine and TRIS spectra were obtained on the chemicals as received (A). Chemical structures showing labeling of the ^12^C atoms as specified in the spectra (B).

The enhancement of *G*_a_ when CuPDA coatings are used as adhesive interlayers could not be explained by the TRIS or Cu^2+^ content in the coatings. We think that the enhanced absorption at ∼1500 and ∼1300 cm^–1^ observed in the IR spectra of CuPDA (see Figures 5 and 6) could provide additional insights in the molecular structure of the coating. Unfortunately, FTIR cannot provide detailed information on the origin of the increased absorption due to substantial peak overlap. Hence, solid-state ^13^C NMR spectroscopy was performed to shed more light on the influence of copper ion during the polymerization of dopamine. The NMR measurements were performed on TiO_2_ nanoparticles (NPs) coated with the PDA and CuPDA coatings used for the adhesion tests shown in Figure 7. The choice for using PDA and CuPDA-coated TiO_2_ NPs instead of just the precipitates from the respective solutions is motivated by the previously reported building block variations between PDA films and PDA precipitates.^38^ In addition, for comparison, solid-state NMR spectra were obtained from as received TiO_2_ NPs, dopamine, TRIS and buffer. The resulting ^13^C solid-state NMR spectra are shown in Figure 8A and the corresponding peaks are assigned according to previous studies.^8,39,40^ For simplicity we assume five chemical species (Figure 8B,a–e) to be present in the PDA and CuPDA coatings.

The ^13^C solid-state NMR spectra obtained from bare TiO_2_ NPs indicate the presence of minor surface contaminants that would not influence spectra obtained from TiO_2_/NPs coated with PDA. PDA coatings produced using 10 mM TRIS-, 50 mM TRIS- and 100 mM bicine buffer exhibit no significant differences in their spectra and are in good agreement with those that are reported in literature for PDA precipitates.^8^ Conversely, spectra obtained from CuPDA coatings vary significantly from the ones obtained from PDA. To begin with, CuPDA coatings produced using TRIS buffer exhibit a peak at ∼60 ppm as well as a “shoulder” at ∼170 ppm that are resonances not present in the CuPDA coatings produced with bicine. The peak at ∼60 ppm corresponds to the C_12_ carbon indicated in Figure 8B and marks the incorporation of TRIS buffer in the PDA structure. The peak at ∼170 ppm corresponds to carbonyl groups that have been reported to be present in PDA either in the form of quinone^8^ or as pyrrolecarboxylic acid^40^ groups. The most notable difference between PDA and CuPDA is observed in the peaks located at 30–40 ppm. In PDA these peaks are assigned to the aliphatic carbons of uncyclized dopamine species (C_13_, C_14_ carbons pointed in Figure 8B). In CuPDA coatings, regardless of the buffer used to produce them, these peaks become broadened and exhibit higher relative intensities. This broadening could be explained by the fact that Cu^2+^ is a paramagnetic relaxation agent, thus causing damping and broadening of NMR peaks, potentially leading to some fully disappearing from the spectrum.^41^ Furthermore, given the different relaxation times, the intensities can also be affected. However, this effect is considered to occur throughout the entire chemical structure, and thus quantitative comparisons are not possible.

The enhanced relative intensity at ∼60 ppm observed for CuPDA with respect to PDA could be accounted to two factors: (1) the hindrance of primary amine cyclization as a result of the complexation of Cu^2+^ and primary amines; or (2) the formation of aliphatic carbon species occurring only when DA polymerizes in the presence of Cu^2+^. Even though both cases are possible based on the FTIR spectra analysis of Figures 5 and 6, the results do not allow to draw definitive conclusions. Finally, we note that PDA coatings produced using 50 mM TRIS did not exhibit any peak at ∼60 ppm which would correspond to TRIS, indicating that TRIS is not present. This is also supported by the atomic concentrations quantified with XPS for PDA coated titanium wires (see SI-9), and contradicts the FTIR results performed in the corresponding PDA precipitates (Figure 6). It is not clear why TRIS appears to be incorporated in PDA precipitates and not in films. We propose that this could be related to the effect of the substrate (titanium in this case) on the PDA polymerization process, as TRIS was incorporated in PDA coatings that were formed on halloysite nanotubes,^42^ even when using 10 mM TRIS and 2 mg mL^–1^ dopamine.

Overall, ^13^C solid-state NMR indicated that (1) the chemistry of CuPDA differs from PDA as indicated by the intensity aliphatic region of the spectra and enhanced carbonyl peaks and (2) CuPDA coatings produced on the surface of titanium using 50 mM TRIS incorporated the buffer in the PDA structure while this was not the case for PDA.

The presence of Cu^2+^ during the formation of PDA resulted in three main variations in the coatings when compared to PDA. These include: (a) the incorporation of Cu^2+^ ions, (b) the incorporation of buffer molecules in the PDA structure (when TRIS is used) and (c) an increased content of aliphatic carbon species. This information is useful to understand why CuPDA enhances bonding between titanium and PC, while PDA does not. We note that the presence of Cu^2+^ during PDA formation has been reported to have no significant impact on the surface morphology of the coatings,^43^ hence in our analysis we focus on changes induced in the chemistry of the coatings. Cu^2+^ ions as indicated by comparing codeposition and postdeposition of Cu^2+^ containing PDA coatings do not appear to play an important role in promoting adhesion between titanium and PC (see Figures 4A and 7). This is further supported by the significant difference in the Cu^2+^ loading between CuPDA produced using bicine (∼1%) and TRIS (∼2.5%) while both coatings resulted in similar *G*_a_ values. The incorporation of TRIS in CuPDA also does not appear to influence adhesion, as CuPDA coatings produced with bicine show no significant variation in adhesion. A notable factor that appears to be important for the enhanced *G*_a_ values observed for CuPDA coatings is the presence of aliphatic carbon species, not found in PDA. The presence of such species is indicated by both the enhanced absorption at ∼1500 and ∼1300 cm^–1^ in FTIR spectra and by a broadened peak of enhanced intensity at 30–40 ppm in ^13^C solid-state NMR spectra. If the data would be interpreted as an increased content of primary amine groups in the CuPDA films, then the previously reported aminolysis of carbonate bonds in PC melts in the presence of primary amines^27^ could explain the enhanced adhesion between CuPDA and PC. However, the identification of the aliphatic species present in CuPDA films (and not PDA) was not possible and consequently the molecular bonding mechanism that occurs between PC and CuPDA during the comolding process remains elusive.

### 3.4 Overview of Metal Ion (M^+^)-Containing PDA in This Work

Metal ion-containing PDA is not a new concept, as it has been utilized in a broad range of applications in the form of coatings or nanoparticles.^13^ However, despite the extensive application of M^+^ containing PDA, understanding of the formation process–structure–properties/functionality relationship is rather limited. In this work we have focused on the application of metal ion-containing PDA coatings formed via a post- or a codeposition process in polymer–metal composites. Here we aim at highlighting the findings related to the applied coatings, beyond the context of polymer–metal adhesion.

Fe^3+^, Fe^2+^, Co^2+^, Ni^2+^, Cu^2+^ and Zn^2+^ ion-containing PDA layers were formed using a postdeposition process (see Figure 1A). The incorporation of Fe^3+^, Co^2+^, Ni^2+^, Cu^2+^ and Zn^2+^ from aqueous solutions did not impact the morphology of the coatings. However, inclusion of Fe^2+^ resulted in the formation of “nanospikes” on the surface of PDA (see Figure 3B). In addition, the M^+^ content in M^+^@PDA was found to be low (typically in the range of 0.3–3%), except for Co^2+^ which surprisingly resulted in contents of ∼12%. This value is high for M^+^@PDA films^13^ and potentially indicates an affinity of PDA for Co^2+^. Another noteworthy feature is that the stability of PDA coatings immersed in 50 mM Fe^3+^ aqueous solutions depends on the type of substrate. PDA coatings formed on SiO_2_ substrates were detached while those formed on TiO_2_ substrates were stable (please refer to SI-10 for further details). PDA coatings immersed in 50 mM Fe^2+^ aqueous solutions were found to be stable at both TiO_2_ and SiO_2_ substrates. This signifies the importance of the substrate-coating interaction when applying M^+^PDA.

Cu^2+^ ion-containing PDA layers were also formed on titanium by using a codeposition process. The codeposition process (see Figure 1A) of Cu^2+^ and PDA, in agreement with previous reports,^13^ provided relatively good control over the Cu^2+^ content of the films. Additionally, we could achieve higher Cu^2+^ loadings compared to the post deposition process. Furthermore, the Cu^2+^ loading in CuPDA was found to be higher in coatings prepared using TRIS than that in bicine buffer. We attribute this to the incorporation of TRIS in CuPDA. TRIS evidently also acts as a coordination site for Cu^2+^, thus enhancing the Cu^2+^ content of the coatings. It is also noted that when high TRIS/DA ratios^40^ are used to produce PDA, TRIS is known to incorporate in the PDA structure. However, even though we observed TRIS incorporation for PDA (2 mg mL^–1^ DA and 50 mM TRIS) precipitates, TRIS was not incorporated in similar PDA films formed on the surface of TiO_2_ NPs or Ti wires. Hence, the presence of Cu^2+^ during DA polymerization could also promote the incorporation of TRIS in PDA layers formed on titanium or similar substrates. This could add more degrees of freedom to the molecular design of PDA, including more possibilities for functionalization of surfaces. Lastly, we showed that the formation of PDA in the presence of Cu^2+^ (CuCl_2_) alters the chemistry of the coatings, in addition to the incorporation of Cu^2+^ or the buffer (in the case of TRIS). Differences between PDA and PDA formed in the presence of Cu^2+^ (CuSO_4_) have been previously indicated by Ball and co-workers^43^ using UV–vis measurements. In the particular study the differences were attributed to local changes induced to 5,6-dihydroxyindole branching in the presence of Cu^2+^.^43^ In our work, ^13^C solid-state NMR measurements indicated that the chemistry of PDA and CuPDA differs due to an increasing concentration of aliphatic carbon species in CuPDA. We do note though that Ball and co-workers^43^ produced CuPDA coatings in acidic conditions (without a buffer) and in the absence of oxygen. In our work CuPDA coatings were produced in the presence of oxygen and by using buffers to stabilize pH at 8.5. Hence a direct comparison could be proven to be misleading and due the lack of reports on the molecular characterization of CuPDA it is not possible to provide more arguments. Additionally, Han and co-workers^44^ using UV–vis measurements reported that the presence of Mg^2+^ and Co^2+^ during the oxidative polymerization of DA influences the formation of intermediate species in PDA, while this was not the case for Na^1+^ and Ca^2+^. The bonding geometry and complex formation constants between catechols and various M^+^ as a function of stoichiometry and pH^45,46^ could potentially explain the aforementioned observations. However, to date these parameters have not been linked to the formation and properties of M^+^PDA films in an in-depth study. This knowledge gap highlights the need for a more systematic investigation on the PDA formation in the presence of M^+^.

## 4 Conclusions

Metal ion-containing polydopamine films were utilized to enhance adhesion between titanium and a PC matrix joined via a comolding process. The films were deposited on the surface of titanium before the joining process using two deposition methods, i.e., postdeposition and codeposition. Fe^3+^, Fe^2+^, Co^2+^, Ni^2+^, Cu^2+^ and Zn^2+^ containing polydopamine films were formed using the post deposition process. AFM measurements indicated that the surface morphology of M^+^PDA coatings remained unchanged compared to PDA, except for Fe^2+^PDA where nanospikes were found to form on the surface. When it comes to promoting adhesion between titanium and PC, Fe^2+^PDA and Fe^3+^PDA resulted in a notable increase in the energy of adhesion (*G*_a_), while the rest of the coatings showed no significant improvements. With the intention to incorporate more Cu^2+^ in PDA, CuPDA coatings were formed on titanium using a codeposition process. The codeposition process was found to alter the chemistry of the coatings beyond the incorporation of Cu^2+^, as indicated by an increased content of aliphatic carbon species compared to PDA. In terms of PC-titanium adhesion, by increasing the Cu^2+^:DA ratio used to produce CuPDA, an increasing trend in *G*_a_ was observed until a ratio of 1:1 above which *G*_a_ remained unchanged. The enhanced adhesion was not related to the presence of Cu^2+^ or TRIS in CuPDA and hence is likely related to the enhanced presence of unidentified aliphatic carbon groups in CuPDA. Overall, through the application in polymer–metal composites, the present work highlights the potential of metal ion-containing PDA coatings to tailor interactions at functional interfaces.

## Supporting Information Available

Fiber pullout results, AFM images of Ti and SiO2 wafers, XPS spectra, and energy of adhesion values (PDF)
