# Supplementary material for: Metal Ions in Polydopamine Coatings Enhance Polymer–Metal Adhesion
Source: ACS Appl Polym Mater. 2025 Feb 12;7(4):2408–18. doi: 10.1021/acsapm.4c03551 (PMC11877418; doi:10.1021/acsapm.4c03551)
Supplement: Supplementary file 1 — ap4c03551_si_001.pdf [file ap4c03551_si_001.pdf]

Supporting Information

Metal ions in polydopamine coatings enhance polymer-metal adhesion

Georgios Kafkopoulos,[a,b] Ricardo P. Martinho,[c] Clemens J. Padberg,[b] Joost Duvigneau,[b] Frederik R. Wurm,[b] and Gyula Julius Vancso*,[a,b]

[a]Department of Materials Science and Technology of Polymers (MTP), University of Twente, Enschede 7522 NB, the Netherlands.

[b]Sustainable Polymer Chemistry (SPC), Department of Molecules and Materials, MESA+ Institute for Nanotechnology, Faculty of Science and Technology, University of Twente, Enschede 7522 NB, the Netherlands.

[c]Department of Molecules and Materials, MESA+ Institute for Nanotechnology, Faculty of Science and Technology, University of Twente, 7500 AE Enschede, The Netherlands

*Corresponding Author
*E-mail: g.j.vancso@utwente.nl (G.J.V.).

SI-1. Pullout testing and analysis

Figure S1A shows a typical pullout curve of a titanium wire - PC pullout sample, schematically represented in Figure S1B. The force is increasing linearly with the crosshead standard travel until a maximum value is reached. In this linear part of the pullout curve crack propagation is initiated (Figure S1A, point "b") and progresses until the maximum force value (F_s), where interfacial detachment is complete and the wire moves with respect to the PC matrix for the first time (i.e. the slip point). After the slip point a sudden drop in the force is observed and then the wire is progressively removed from the PC matrix while maintaining frictional contact.

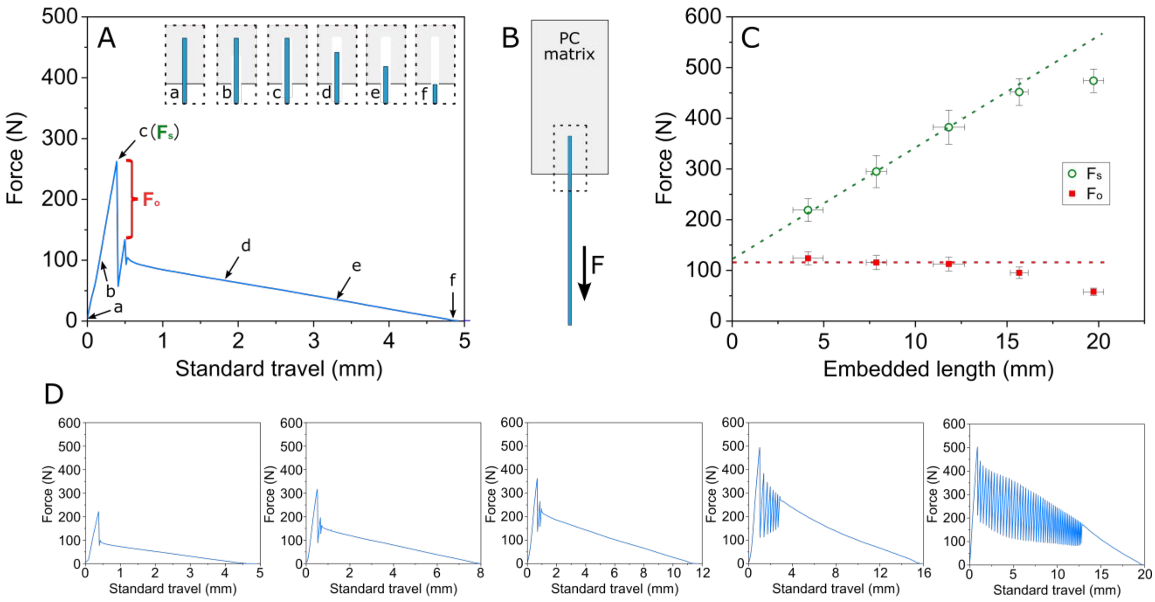

Figure S1. Typical pullout curve of a titanium wire from a PC matrix (A); the schematic insets represent the stages of the pullout process and are assigned to their corresponding part of the curves (a-f); The F_s and F_o notations correspond to the slip force and the force required to maintain crack propagation in zero friction conditions respectively. Schematic representation of a pullout specimen (B); the dashed rectangle indicates the corresponding area of the insets in panel A. F_o and F_s values versus the embedded length of the titanium wire in the PC matrix (C). Typical pullout curves for each embedded length value of plot C (D).

As we have argued in our previous work,[1] the interfacial energy of adhesion (G_a) represents the energy per surface area needed to cause interfacial detachment and can be calculated using the following equation[2]:

$$G_a = {F_o}^2(4\pi^2 r^3 E_f)^{-1} \quad \text{Eq. 1}$$

where, F_o is the debonding force required to maintain crack propagation in zero friction conditions,[3] r is the radius and E_f the modulus of the wire. E_f and r are known values and F_o can be estimated either from the pullout curve (see Figure S1A).

To validate that the analysis[3] used to obtain Eq. 1 is applicable for our pullout system, the F_o and F_s values are plotted for various embedded lengths (L_e). According to the particular analysis, F_s follows a linearly increasing trend with L_e while F_o is independent of L_e, at low L_e values. Figure S1C show the F_s and F_o values obtained for various embedding lengths of titanium wires in the PC matrix and validates the predictions of the analysis[3] used to obtain Eq. 1. In addition, the extrapolation of F_s and F_o to zero embedded length results in similar force values, which also falls within the predictions of the aforementioned analysis. Finally for F_s values above 400 N, F_o values are no longer independent of the embedded length. Thus, G_a values obtained from plots with an F_s above 400 N are expected to be underestimated.

SI-2. M^+ post deposition F_o values & pullout curves

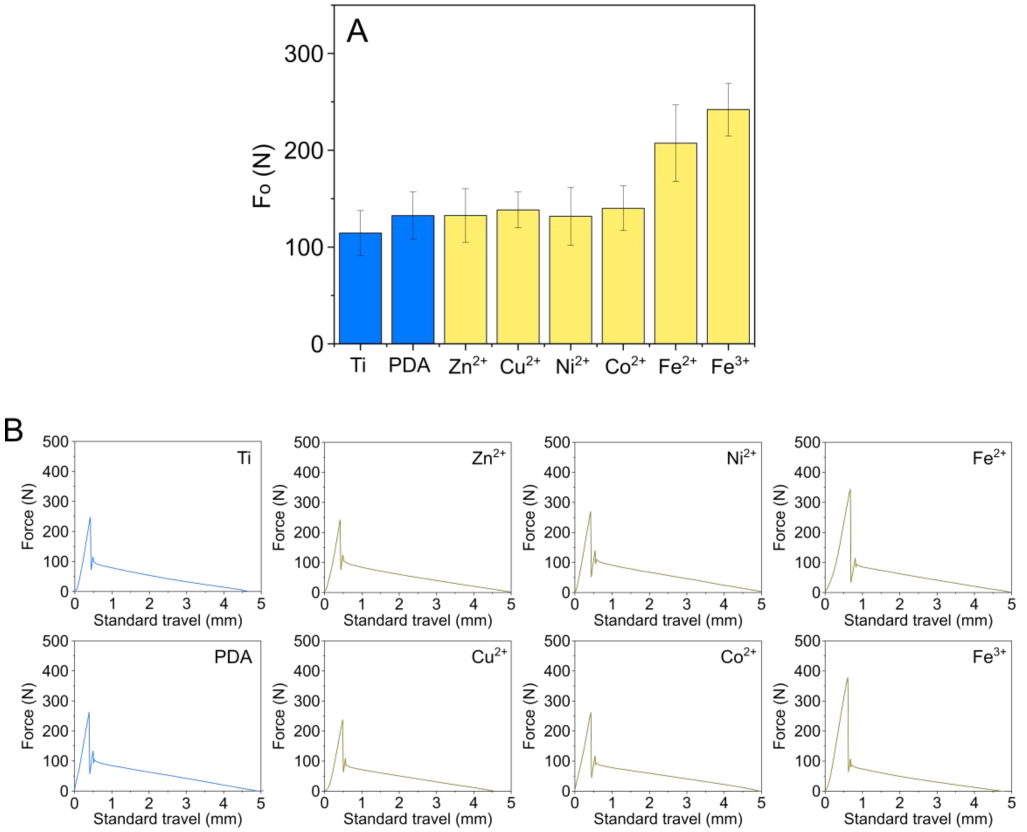

Figure S2. F_o values determined from the pullout curves of specimens comprising of PC and unmodified, PDA or M^+PDA coated titanium wires (A) and representative pullout curves for each sample category shown in plot A (B).

SI-3. AFM Height images of M^+@PDA coated Ti@SiO_2 wafers

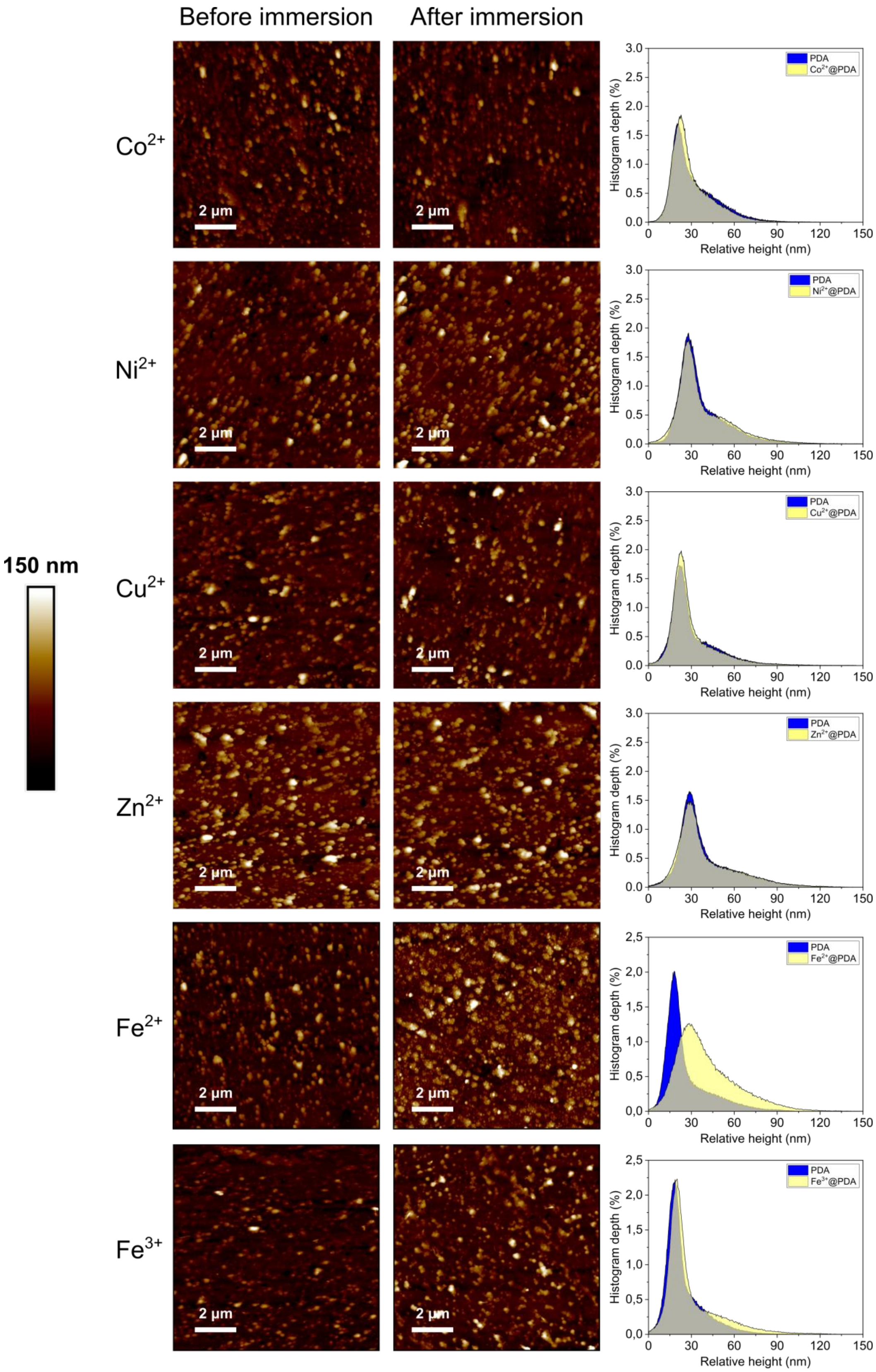

Figure S3. AFM height maps of PDA coatings deposited on the surface of titanium coated SiO_2 wafers before and after their immersion in M^+ aqueous solutions with their corresponding height distributions (0 nm height corresponds to the lowest point of the surface of the coatings).

SI-4. XPS Spectra & atomic concentrations of M^{+}PDA coatings

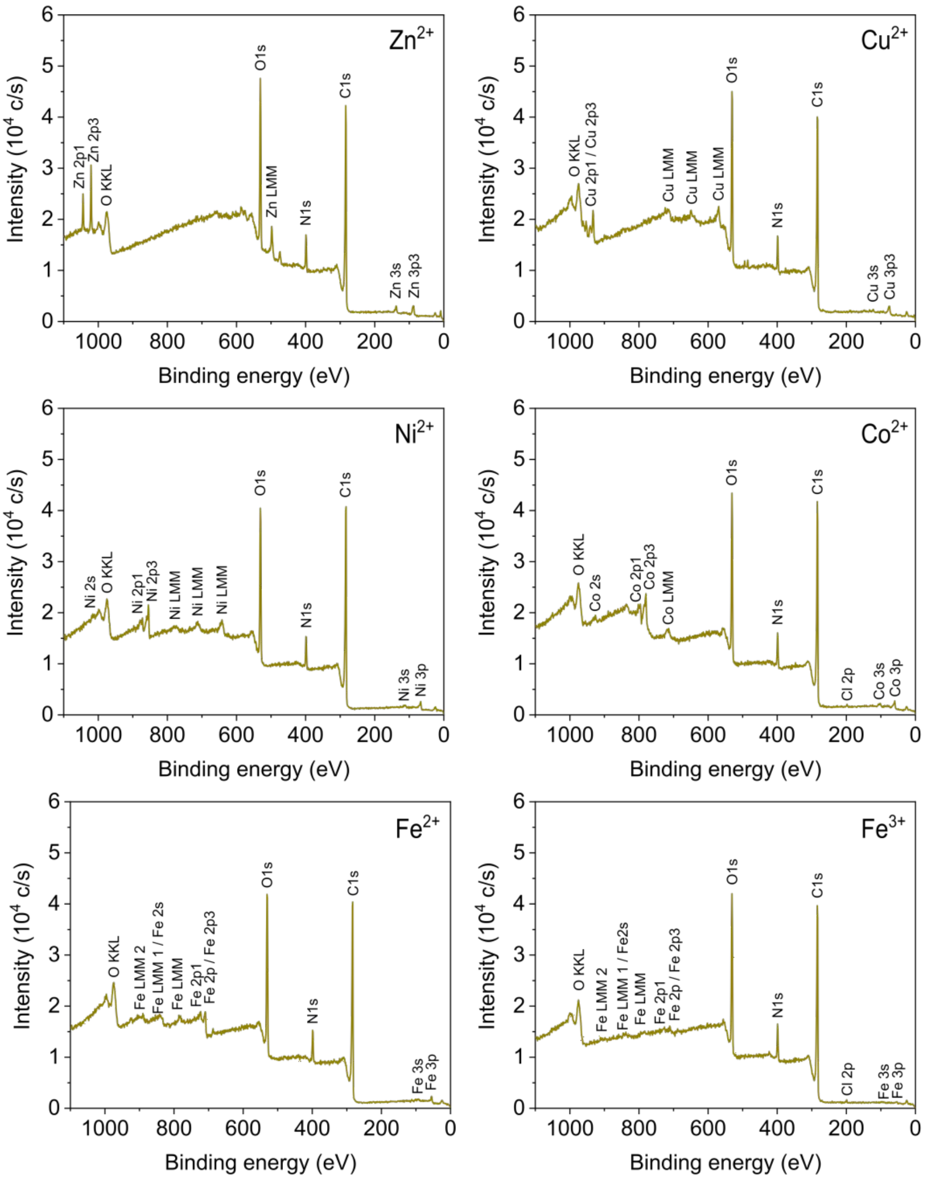

Figure S4. XPS spectra obtained from M^{+}PDA coatings deposited on the surface of titanium wires.

Table S1. Atomic concentrations determined from the XPS spectra of Figure S4.

	C	N	O	Cl	M^{+}
**Fe^{3+}@PDA**	72,12 ± 1,42	6,62 ± 0,68	20,57 ± 1,13	0,32 ± 0,04	0,37 ± 0,22
**Fe^{2+}@PDA**	57,31 ± 5,01	3,78 ± 1,45	34,68 ± 6,64	-	3,53 ± 0,73
**Co^{2+}@PDA**	53,37 ± 4,65	4,68 ± 0,92	27,67 ± 1,12	1,99 ± 0,55	12,29 ± 3,85
**Ni^{2+}@PDA**	70,70 ± 2,06	6,31 ± 0,49	21,35 ± 1,17	-	1,67 ± 0,66
**Cu^{2+}@PDA**	68,17 ± 1,24	6,28 ± 0,38	23,75 ± 0,86	-	1,75 ± 0,27
**Zn^{2+}@PDA**	70,75 ± 1,34	6,46 ± 0,36	21,44 ± 0,79	-	1,35 ± 0,59

SI-5. M^+ co-deposition

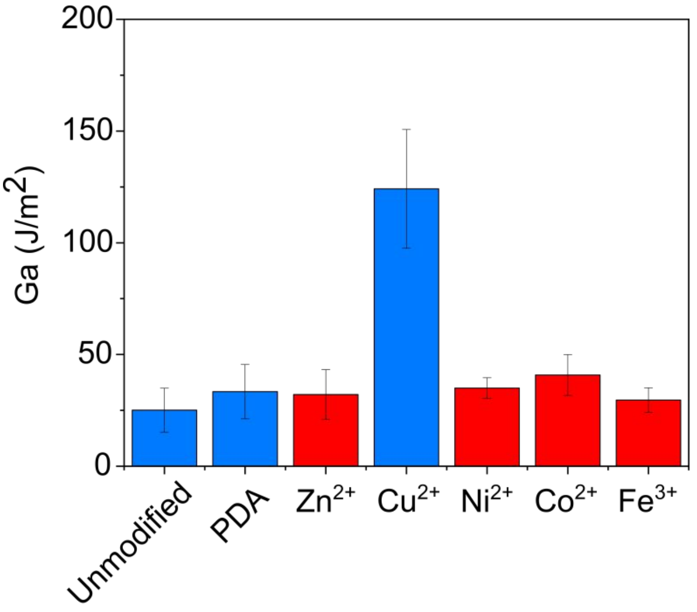

Figure S5. Energy of adhesion values between PC and unmodified, PDA and MPDA (co-deposition of DA and M^+ at a 1:1 M^+:DA molar ratio) coated titanium wires, determined by pullout tests. Red bars represent trials of 2 samples per category, while blue bars represent a complete data set with at least 6 samples per category.

SI-6. CuPDA co-deposition F_o values & pullout curves

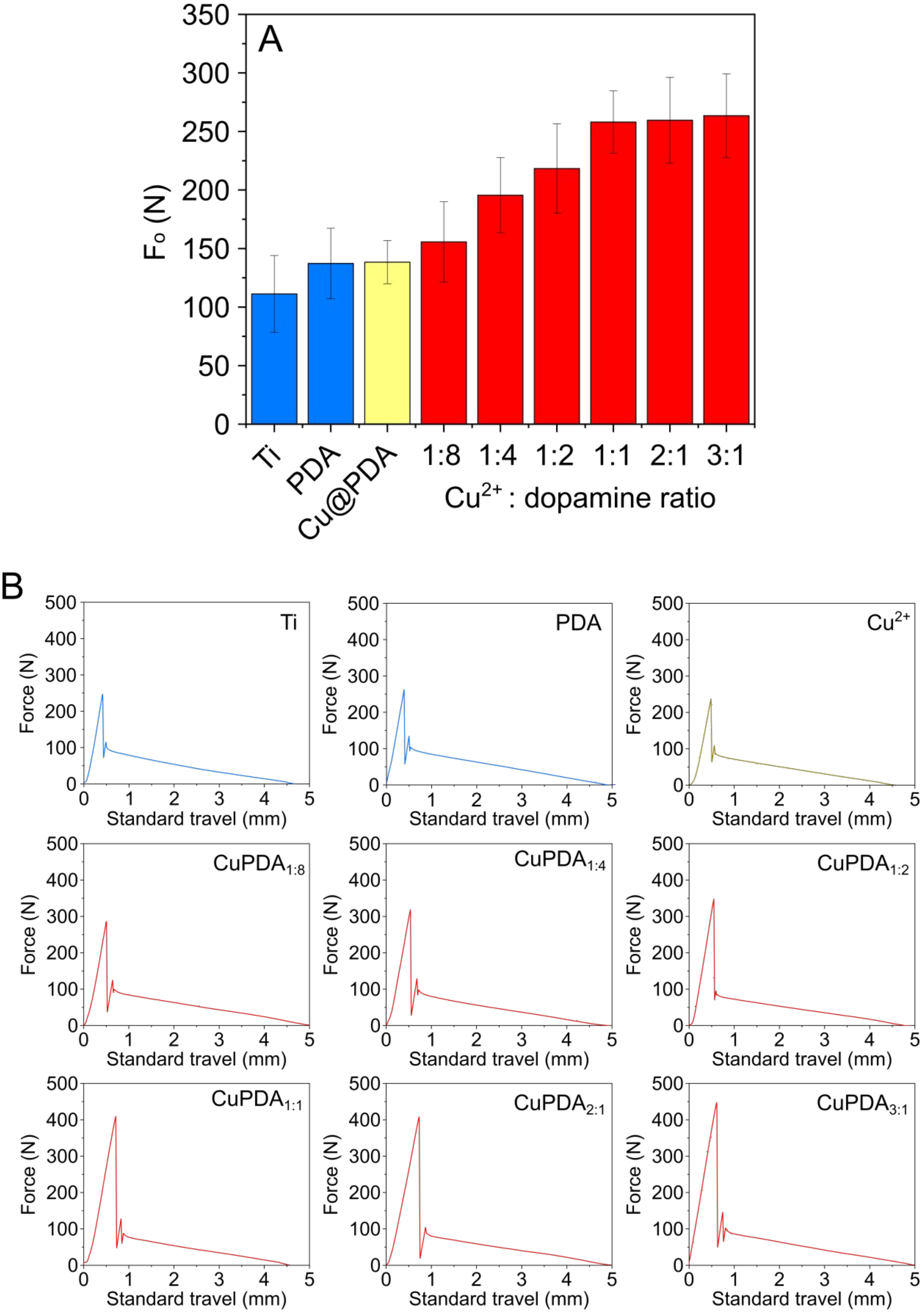

Figure S6. F_o values determined from the pullout curves of specimens comprising of PC and unmodified, PDA, Cu^{2+}@PDA or CuPDA coated titanium wires (A) and representative pullout curves for each sample category shown in plot A (B).

SI-7. XPS Spectra & atomic concentrations of $M^{+}PDA$ coatings

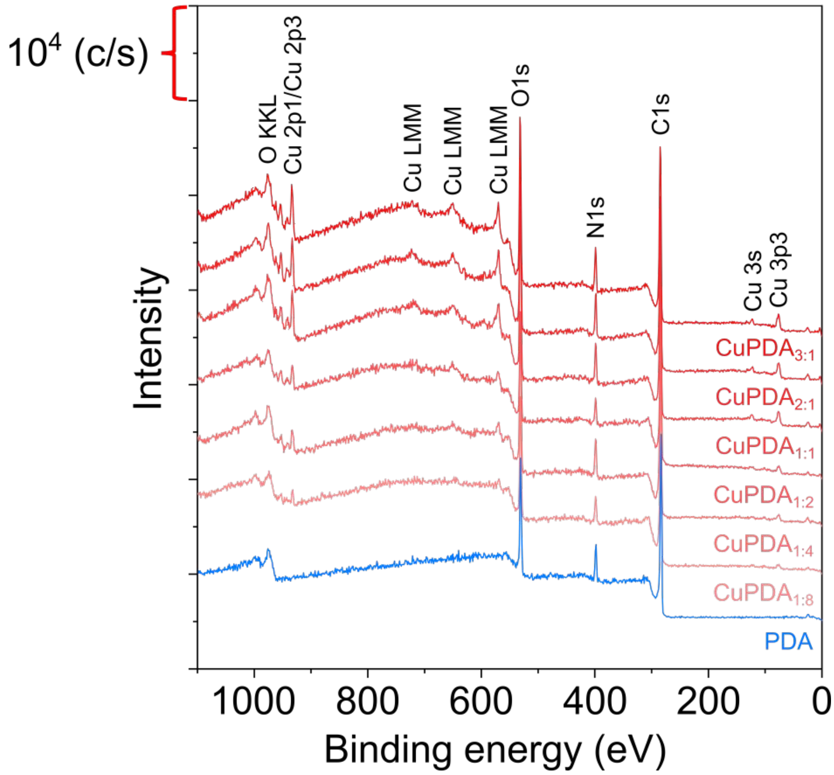

Figure S7. XPS spectra obtained from PDA and CuPDA coatings deposited on the surface of titanium wires.

Table S2. Atomic concentrations determined from the XPS spectra of Figure S7.

	**C**	**N**	**O**	**Cu^{2+}**
**PDA**	72,36 ± 0,45	8.44 ± 0,59	19,20 ± 0,16	-
**$CuPDA_{1:8}$**	75,73 ± 0,54	5,93 ± 0,31	17,70 ± 0,68	0,64 ± 0,16
**$CuPDA_{1:4}$**	73,69 ± 1,51	6,13 ± 0,61	18,71 ± 1,09	1,08 ± 0,21
**$CuPDA_{1:2}$**	75.63 ± 1,49	6,11 ± 0,82	16,89 ± 0.93	1,34 ± 0,23
**$CuPDA_{1:1}$**	66,69 ± 1,49	7,44 ± 1,26	23,30 ± 0,33	2,57 ± 0,12
**$CuPDA_{2:1}$**	64,21 ± 0.43	8,49 ± 0,32	24,41 ± 0,21	2,88 ± 0,30
**$CuPDA_{3:1}$**	64,23 ± 0,61	7,95 ± 0,30	24.49 ± 0,29	3,33 ± 0,30

SI-8. XPS Spectra & atomic concentrations of coatings used for the control experiment

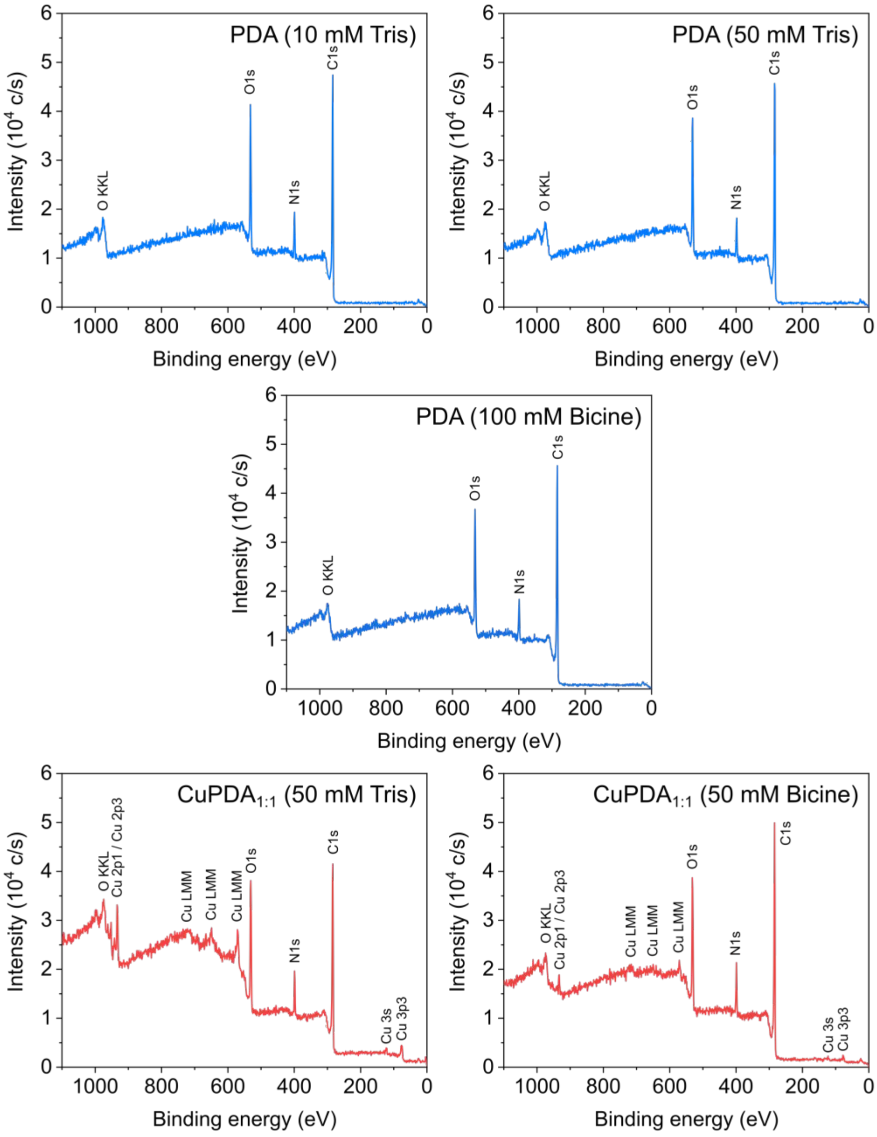

Figure S8. XPS spectra obtained from PDA and CuPDA coatings deposited on the surface of titanium wires. The coatings differ on the buffer and buffer concentration used for the deposition process.

Table S3. Atomic concentrations determined from the XPS spectra of Figure S8.

	C	N	O	Cu^{2+}
**PDA 10 mM Tris**	72,36 ± 0,45	8.44 ± 0,59	19,20 ± 0,16	-
**PDA 50 mM Tris**	72,79 ± 0,48	8,37 ± 0,94	18,84 ± 0,63	-
**PDA 100 mM Bicine**	74,43 ± 1,44	7,73 ± 0,55	17,95 ± 1,19	-
**$CuPDA_{1:1}$ 50 mM Tris**	66,69 ± 1,49	7,44 ± 1,26	23,30 ± 0,33	2,57 ± 0,12
**$CuPDA_{1:1}$ 100 mM Bicine**	70,94 ± 0.67	8,57 ± 0,28	19,48 ± 0,59	1,01 ± 0,09

SI-9. PDA and CuPDA control experiment F_o values & pullout curves

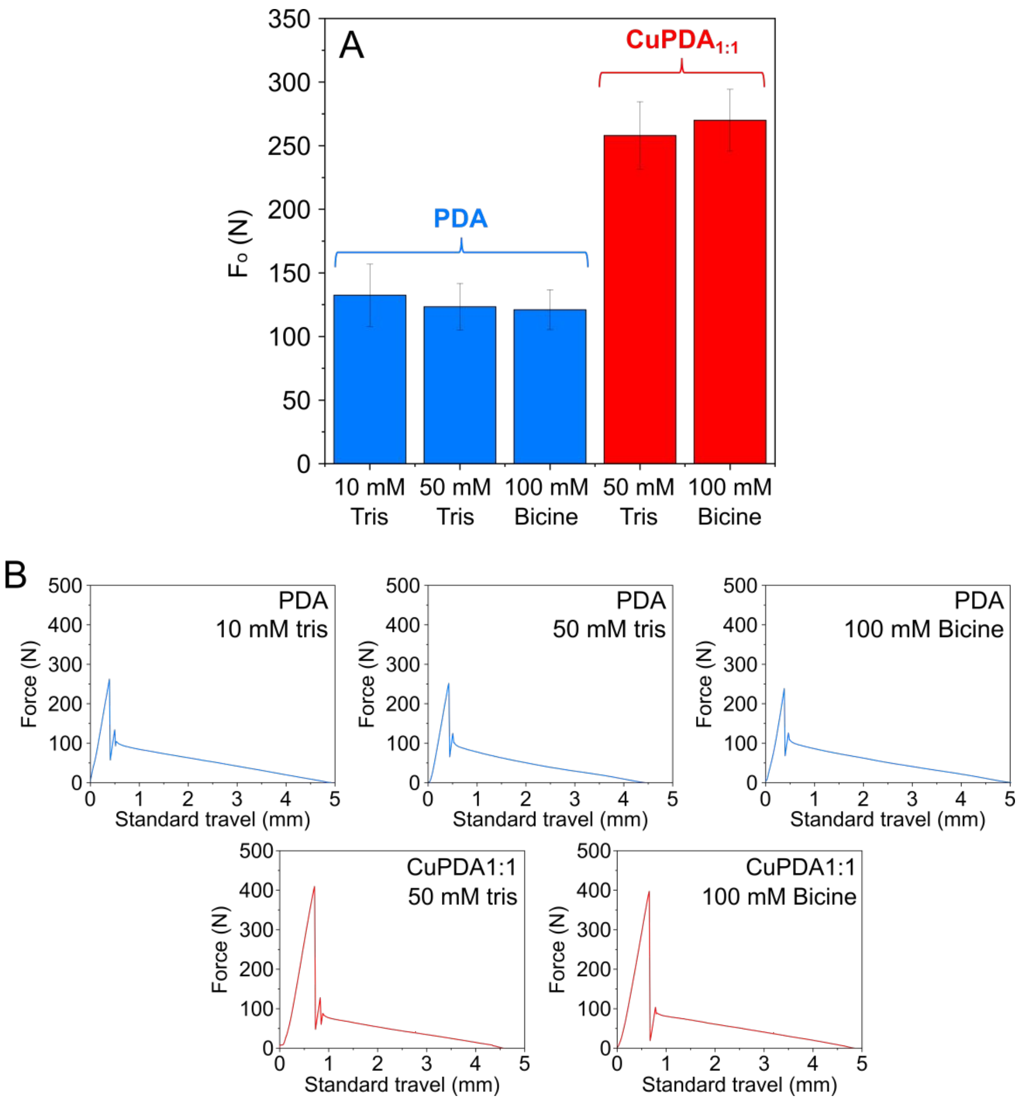

Figure S9. F_o values determined from the pullout curves of specimens comprising of PC and PDA or CuPDA coated titanium wires; the PDA and CuPDA coatings vary on the type and concentration of buffer used to synthesize them (A). Representative pullout curves for each sample category shown in plot A (B).

SI-10. Stability of PDA coatings formed on SiO_2 and TiO_2 substrates

TiO_2 and SiO_2 substrates coated with ~80 nm thick PDA layers were immersed halfway in vigorously stirring Fe^{2+} or Fe^{3+} aq. solutions (see Figure S10A). This experimental procedure was used to evaluate whether the coatings can survive the post deposition process when formed on different substrates. The wafers were prepared according to section 2.4 of the main article and were stored in water until they were used for the stability tests. The results shown in Figure S10B and C suggest that all testing groups were proven stable except for PDA formed on SiO_2, which detaches after immersion at Fe^{3+} aq. solutions. This is potentially the result of the nature of interaction of PDA layers and different substrates, i.e. coordination bonds for TiO_2 and secondary interactions for SiO_2,[4] in combination with the competition introduced by the complexation of $Fe^{3+}$with catechol groups in PDA. This would weaken the interaction between coating and substrate, resulting in immediate detachment after immersion in the Fe^{3+} aq. solution.

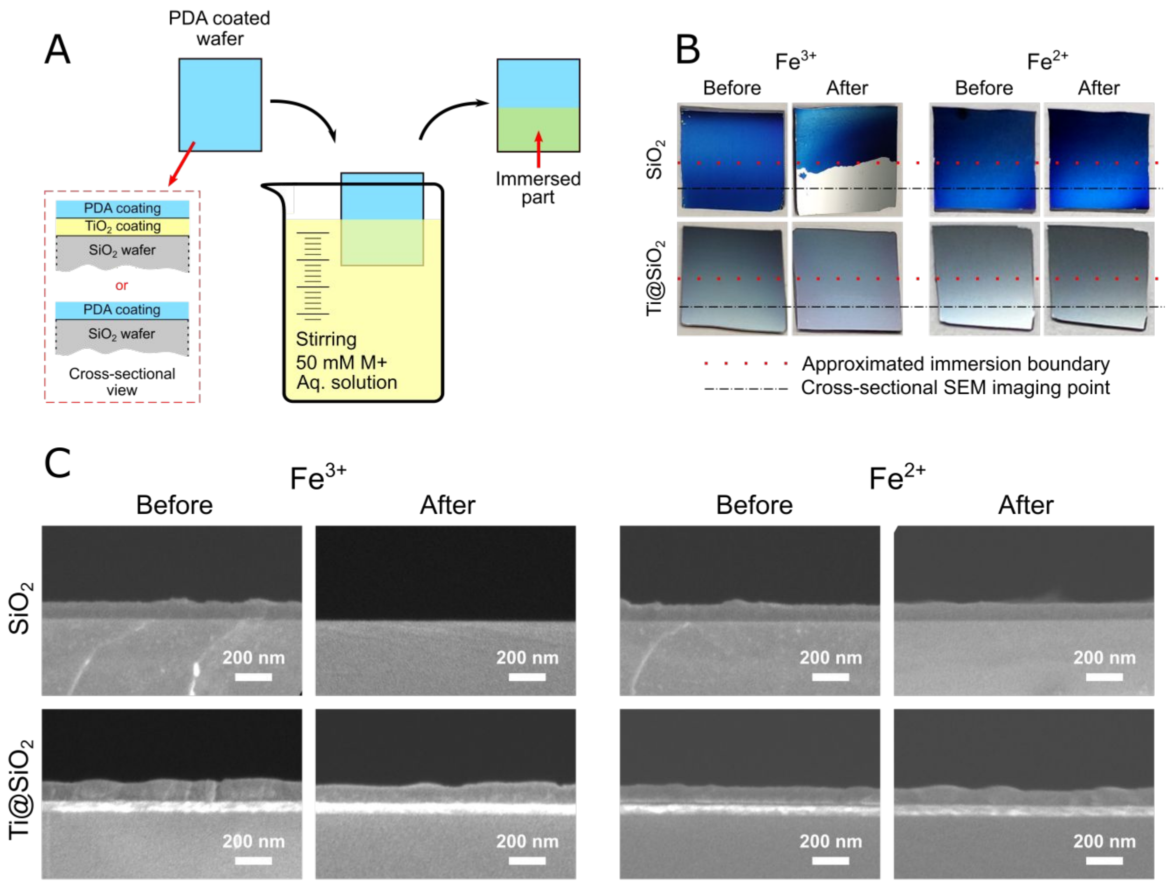

Figure S10. Schematic representation of the experimental process used to evaluate the stability of PDA coatings deposited on SiO_2 and TiO_2 substrates after immersion in 50 mM Fe^{2+} or Fe^{3+} solutions (A). Photographs taken in an angle of ~ 45° of the respective samples before and after the immersion process (B) and cross-sectional SEM imaging of the respective samples before and after the immersion process (C).

References

(1) Kafkopoulos, G.; Padberg, C. J.; Duvigneau, J.; Vancso, G. J. Adhesion Engineering in Polymer-Metal Comolded Joints with Biomimetic Polydopamine. *ACS Appl. Mater. Interfaces* **2021**, *13* (16), 19244–19253. https://doi.org/10.1021/acsami.1c01070.

(2) Gray, R. J. Analysis of the Effect of Embedded Fibre Length on Fibre Debonding and Pull-out from an Elastic Matrix - Part 2 Application to a Steel Fibre-Cementitious Matrix Composite System. *J. Mater. Sci.* **1984**, *19* (5), 1680–1691. https://doi.org/10.1007/BF00563066.

(3) Wang, C. Fracture Mechanics of Single-Fibre Pull-out Test. *J. Mater. Sci.* **1997**, *32* (2), 483–490. https://doi.org/10.1023/A:1018534323464.

(4) Lee, H.; Hong, S.; Park, H. K.; Park, J. H. Material-Selective Polydopamine Coating in Dimethyl Sulfoxide. *ACS Appl. Mater. Interfaces* **2020**, *12* (43), 49146–49154. https://doi.org/10.1021/acsami.0c11440.
